# Lancereaux, diabète maigre, and diabète gras revisited

**DOI:** 10.1177/09677720241267058

**Published:** 2024-08-21

**Authors:** James R. Wright, Lynn McIntyre

**Affiliations:** Department of Pathology and Laboratory Medicine, Cumming School of Medicine, University of Calgary, Calgary, Alberta, Canada; Department of Community Health Sciences, Cumming School of Medicine, University of Calgary, Alberta, Canada

We were surprised to be notified of a new citation (“Diabète Maigre and Diabète Gras”^1^) to our paper, “Misread and mistaken: Étienne Lancereaux's enduring legacy in the classification of diabetes mellitus”^2^ without having been offered the opportunity to respond to the critique. We are responding now.

Connor and Boulton's general premise is that one must be wary of pitfalls inherent in retrospective medical diagnoses, citing a paper by former *Journal of Medical Biography* (JMB) editor AJ Larner.^1^ We agree that there are inherent issues with modern-day clinicians assigning specific medical diagnoses to specific historical figures based upon symptomatology. Larner was the editor of JMB at the time our paper was submitted and reviewed. Since he did not raise any such concerns during our paper's peer-review process, it is likely that he did not believe that it applied to our paper which discusses three highly cited, but seldom read, late nineteenth-century French papers describing clinicopathological findings in two series of diabetic patients. While we fully agree with Connor and Boulton that nosology, terminology, and conceptualization of disease can change significantly over time, the fundamental pathological and biological properties of diabetes mellitus (DM) cannot have changed profoundly in the past 140 years.

Connor and Boulton seem preoccupied with our discussion of Lancereaux's “histological” findings. They state that “the reader is dependent on the current authors’ translation and interpretation of selected extracts from the French text” because “the original histology on the two autopsies is not available.”^1^ This criticism could be made for all historical reviews of histological studies at that time because the earliest method of preparing tissues for microscopic study was by cutting and staining frozen sections, and such preparations could not be preserved for more than a few hours until 1895.^3^ However, these criticisms are problematic for another reason, as we had not actually specifically mentioned any of Lancereaux's histological findings in our paper, as they were not as informative as his gross pathological (macroscopic) findings. For instance, Connor and Boulton write: “The histology in Patient 1 is described in two of the original papers but one paper mentions calcification and the other does not.”^1^ Au contraire, we were describing a significant macroscopic discrepancy, not a subtle microscopic one; in 1877, the autopsy findings on this patient make no mention of pancreatic ductal calculi (lithiasis) but when the same patient's autopsy findings are described in 1880, lithiasis is highlighted. Such an error is noteworthy as pancreatic lithiasis was now Lancereaux's proposed cause for cases of thin diabetes (diabète maigre).

In patient 2, we use the term “fibrocalculous disease” to summarize Lancereaux's findings of pancreatic atrophy with interstitial fibrosis associated with pancreatic ductal lithiasis, an entity that would be named “chronic interstitial pancreatitis, interlobular type” in 1901.^4^ And of course, two decades later, Frederick Banting's great idea that initiated the sequence of events resulting in the discovery of insulin would begin with Banting reading a case report about this very pancreatic lithiasis-associated entity.^4,5^ For Connor and Boulton to suggest that this term might refer to a variant of DM first described in 2015 “Fibrocalculous Pancreatic Diabetes, a disease almost entirely restricted to the tropics”^1^ is far-fetched as Lanceraux's clinical history indicates this patient was a Belgian cabinet maker living in Paris rather than an “officer or official in a French colony.”^1^

Next, we were criticized for not mentioning that pancreatic islets had been discovered by Paul Langerhans “in 1869, eight years before Lancereaux's first paper.”^1^ Lancereaux did not mention these structures in his brief histological descriptions because he would have been entirely unaware of their existence—as was the world. Victor Medvie, in his *A History of Endocrinology*, explains that Langerhans described these pale-staining histologic foci scattered throughout the pancreas in his medical student dissertation and thought them so unimportant that he apologized to his dissertation examiners and hoped they “would look tolerantly on his efforts”^5^ and not fail him. In fact, he wondered if they were small lymph nodes. Langerhans and his findings would have been entirely forgotten had not Gustave Edouard Laguesse, while studying fetal pancreatic histology in 1893 (more than a decade after Lancereaux's final paper), somehow stumbled “in some extraordinary way” onto Langerhans dissertation and, “with charity” suggested the eponym islets of Langerhans.^5,6^

As for mentioning the ICD-9 code for Lancereaux diabetes, this was included as we were publishing a paper on Lancereaux in a history journal focused on medical biography; why would we not mention this, even if the terminology is now only historical? If the street in Paris named for Lancereaux were renamed, we would also comment on where it had been. Finally, our mention of type 3c diabetes was added to satisfy one of the paper's peer-reviewers; while perhaps tangential and distracting, the statement as written is true.

Connor and Boulton in their volley of criticisms never attack our seminal observation that Lancereaux's clinical and autopsy studies have been erroneously considered to be documentation that the medical world by 1880 was well aware of two distinct major types of DM, resembling current-day type I and type II DM. As our paper clearly describes^1^ and as reinforced elsewhere,^7^ Lancereaux's ideas had already been fully discounted and then forgotten by the early twentieth century, except for small pockets of regional fans in France and Romania; Lancereaux's ideas were rediscovered, misinterpreted, and then popularized by those superficially interested in the history of diabetes during the current authors’ lifetimes.

While we fully recognize that patients “aged 42, 35, and 35”^1^ are not outside the possible range for age of onset for type I (until fairly recently called “juvenile-onset”) DM, the typical age of onset for DM cases now classified as type I clusters mostly toward a much younger age group while those now classified as type II tend to be middle-aged or older, as were all of Lancereaux's thin patients, including his 61-year-old. Furthermore, it is biologically implausible to suggest that highly emaciated type I diabetic patients uniformly lived for two or three years after diagnosis (or, in one case, Lancereaux's diagnosis was made only 15 days before death but Lancereaux specifically noted that he had first seen this patient and made his diagnosis two years after the patient became fully symptomatic) prior to the discovery of insulin, when even Frederick Allen's and Elliot Joslin's rigorous, meticulous life-prolonging dietary therapies could not accomplish such results in the decade immediately preceding the discovery of insulin.^7^ Some of Lancereaux's thin patients (n.b., he maintained likely all) had pancreatic atrophy secondary to pancreatic calculi, a finding that would exclude a type I diagnosis now; currently, islet β-cell autoimmunity is believed to cause type I DM and the gross appearance of the pancreas at autopsy in type I diabetic patients is generally unremarkable. Therefore, the preponderance of evidence, correctly interpreted >140 years later, fully supports our analysis, and we can safely conclude that Lancereaux did not describe two distinct types of DM that were forerunners of type I and type II DM, nor did his thin diabetic patients have what would now be called type I DM.
